# The Increase in Cancer

**Published:** 1904-05

**Authors:** 


### SELECTION.

### The Increase in Cancer.

(Editorial in *New York Tribune*, April 16, 1904.)

Dr. Roswell Park of Buffalo, while delivering an address in Berlin this week, made an allusion to the prevalence of cancer which has either been misunderstood or inaccurately reported. He is represented as having said that cancer is almost as common in the United States as tuberculosis. This is a matter to which Dr. Park has given much attention for years, and he has made a special study of the conditions in New York State, which is supposed to lie in what is called the "cancer belt." Hence, it does not seem credible that he intended to convey the impression which one would derive from the language imputed to him.

It appears from the statistics of the State Board of Health for 1903, for instance, that the total number of deaths last year from cancer in New York was 5,456, while the mortality was considerably greater from each of seven other diseases, not counting accidents, violence and unclassified maladies. "Acute respiratory disorders," among which pneumonia doubtless holds the chief place, are credited with 17,339 deaths, and tuberculosis with 13,194. From the same source of information, however, one learns that there has been comparatively little fluctuation of late in the havoc from most of these causes, but that cancer is on the increase.

Deaths in 1903 due to cancer exceeded those of 1902 by nearly 500, and exceeded the average for the previous five years by 684. These figures show, therefore, that the rise in mortality from cancer, to which Dr. Park called attention in the pages of the medical papers five years ago, still continues. He then predicted that if it was maintained long enough cancer would rival tuberculosis. That time has not yet arrived, but the steady increase for more than a decade certainly justifies great uneasiness. After all, it is the ratio that tells the story.

Corroborative testimony was offered by Dr. Frank G. Clemow, in his Geography of Disease, which appeared last year. He was disposed to think that to a limited extent, possibly, the gain was apparent rather than real. In some measure it might perhaps be attributed to differences in diagnosis and registration between earlier and later periods, but it did not seem probable to him that any such explanation would fully account for the increase. The phenomenon has been observed in several European countries. It was especially notable in the United Kingdom. Still, the most conspicuous rise, he declared, was on this side of the Atlantic. As to the main fact, therefore, there can be little question. It has been recognised too long and too widely to admit of mistake.

Medical men have at least two reasons for regarding this situation with peculiar dismay. No altogether satisfactory method of treatment for cancer has yet been found. In a few cases Röntgen rays have afforded relief. Perhaps radium will some day prove helpful. Surgery is the most efficacious resource, but it is not always practicable, nor does it invariably preclude a return of the trouble. The wisest and most skilful members of the profession are almost powerless to fight cancer. Again, the exact cause of this disease—or, to speak more accurately, this group of diseases—has not yet been determined. Every few months the alleged discovery of the cancer germ is announced. The men who have been engaged in the hunt and who have thought that they had achieved success have often had a high standing. One of them, Dr. Gaylord, is a fellow townsman of Dr. Park. Another is Dr. Schmidt, one of the advisers of the Emperor of Germany. In none of these instances, however, has the demonstration been complete and convincing. An organism may be found which is almost always an accompaniment of the diseased tissue under examination. It will not meet all the logical requirements prescribed long ago by Koch, though, unless it be separated from the human system, independently cultivated and then employed to transmit disease to a healthy animal. As final proof of that kind is still lacking, it is too soon to place confidence in any of these claims. Eventually, it is reasonable to hope, the mysteries of cancer will be revealed and an effective method of warfare will be found, but today the doctors are sadly in the dark.

